# Fitness and Productivity Increase with Ecotypic Diversity among *Escherichia coli* Strains That Coevolved in a Simple, Constant Environment

**DOI:** 10.1128/AEM.00051-20

**Published:** 2020-04-01

**Authors:** Dong-Dong Yang, Ashley Alexander, Margie Kinnersley, Emily Cook, Amy Caudy, Adam Rosebrock, Frank Rosenzweig

**Affiliations:** a Division Biological Sciences, University of Montana, Missoula, Montana, USA; b Donnelly Centre, University of Toronto, Toronto, Ontario, Canada; c School of Biology, Georgia Institute of Technology, Atlanta, Georgia, USA; University of Illinois at Urbana-Champaign

**Keywords:** chemostat, *E. coli*, cross-feeding, fitness, diversity, productivity, ecotypes, consortia

## Abstract

The productivity of a biological community often correlates with its diversity. In the microbial world this phenomenon can sometimes be explained by positive, density-dependent interactions such as cross-feeding and syntrophy. These metabolic interactions help account for the astonishing variety of microbial life and drive many of the biogeochemical cycles without which life as we know it could not exist. While it is difficult to recapitulate experimentally how these interactions evolved among multiple taxa, we can explore in the laboratory how they arise within one. These experiments provide insight into how different bacterial ecotypes evolve and from these, possibly new “species.” We have previously shown that in a simple, constant environment a single clone of Escherichia coli can give rise to a consortium of genetically and phenotypically differentiated strains, in effect, a set of ecotypes, that coexist by cross-feeding. We marked these different ecotypes and their shared ancestor by integrating fluorescent protein into their genomes and then used flow cytometry to show that each evolved strain is more fit than the shared ancestor, that pairs of evolved strains are fitter still, and that the entire consortium is the fittest of all. We further demonstrate that the rank order of fitness values agrees with estimates of yield, indicating that an experimentally evolved consortium more efficiently converts primary and secondary resources to offspring than its ancestor or any member acting in isolation.

**IMPORTANCE** Polymicrobial consortia occur in both environmental and clinical settings. In many cases, diversity and productivity correlate in these consortia, especially when sustained by positive, density-dependent interactions. However, the evolutionary history of such entities is typically obscure, making it difficult to establish the relative fitness of consortium partners and to use those data to illuminate the diversity-productivity relationship. Here, we dissect an Escherichia coli consortium that evolved under continuous glucose limitation in the laboratory from a single common ancestor. We show that a partnership consisting of cross-feeding ecotypes is better able to secure primary and secondary resources and to convert those resources to offspring than the ancestral clone. Such interactions may be a prelude to a special form of syntrophy and are likely determinants of microbial community structure in nature, including those having clinical significance such as chronic infections.

## Abstract (precis)

Polymicrobial consortia occur in both environmental and clinical settings. In many cases, diversity and productivity correlate in these consortia, especially when sustained by positive, density-dependent interactions. However, the evolutionary history of such entities is typically obscure, making it difficult to establish the relative fitness of consortium partners and to use those data to illuminate the diversity-productivity relationship. Here, we dissect an Escherichia coli consortium that evolved under continuous glucose limitation in the laboratory from a single common ancestor. We show that a partnership consisting of cross-feeding ecotypes is better able to secure primary and secondary resources and to convert those resources to offspring than the ancestral clone. Such interactions may be a prelude to a special form of syntrophy and are likely determinants of microbial community structure in nature, including those having clinical significance such as chronic infections.

## INTRODUCTION

Microbial communities in nature exhibit enormous genetic diversity owing in part to spatial and temporal heterogeneity of resources at the microscopic scale. This heterogeneity opens up ample opportunities for selection and drift to act differentially on new variants arising by mutation or by horizontal gene transfer as well as those arriving via dispersal. Over geologic time scales, these evolutionary forces have enabled microbes to exploit almost every environment on and in the Earth’s crust, partitioning niches according to how they differ in their physiological tolerances and in their electron donor and acceptor preferences (1). Microbes not only partition existing niches, they also create new ones. For example, one microbial taxon may release metabolites that sustain others, who by consuming them relieve product inhibition or render thermodynamically unfavorable reaction sequences favorable (2). Among widely diverged taxa, interspecies transfer of metabolites can promote stable associations that increase the amount of energy extracted from the surrounding environment, a phenomenon known as syntrophy (3).

Over ecological time scales, selection on an initially clonal bacterial population can produce different genetic structures depending on the number and the relative fitness of new adaptive mutants and how those mutants interact. If the number is low and/or large fitness gains are confined to one mutant, then selection tends to favor clonal replacement (periodic selection) of successively fitter adaptive lineages (4, 5). If the number of new adaptive mutants is large, their fitness differences small, and their interactions governed chiefly by exploitative competition, then selection will tend to result in clonal interference (6, 7). However, if multiple adaptive mutations arise and they interact in ways other than exploitative competition, then selection may favor clonal reinforcement (8). This outcome manifests as prolonged coexistence of multiple adaptive lineages supported by frequency-dependent interactions such as differential use of public goods (9), mutual inhibition (10), and/or metabolic cross-feeding (11, 12). These three different outcomes are not mutually exclusive: clonal interference could arise within any of several stably coexisting lineages, as could periodic selection of highly fit novel variants (13). Moreover, adaptive lineages that coexist owing to frequency-dependent selection are more likely to undergo evolutionary diversification than those that are not (14, 15). Over successive generations such lineages may become so genetically and physiologically differentiated as to attain the status of ecotypes (16), especially when barriers to horizontal gene transfer arise between them. It is an open question whether such ecotypes constitute nascent bacterial species, *sensu* Cohan (17), that in time go on to form, for example, stable syntrophic associations.

Helling et al. were among the first to report stable coexistence of multiple genotypes in lab populations of Escherichia coli originating from a single, common ancestor (18). This was surprising given that the evolution experiments were carried out in aerobic, glucose-limited chemostat cultures run at a fixed dilution rate and temperature. All things being equal, frequency-dependent selection should be less impactful in a simple, constant environment than in serially transferred batch cultures (19), a fluctuating environment where evolving populations regularly undergo cycles of feast and famine. Serial transfer cultures have been shown to select for variants adapted to one or the other condition (20–22), especially in mixed resource (23, 24) and alternating resource (25) environments, and these variants can coexist for many generations.

**FIG 1:**
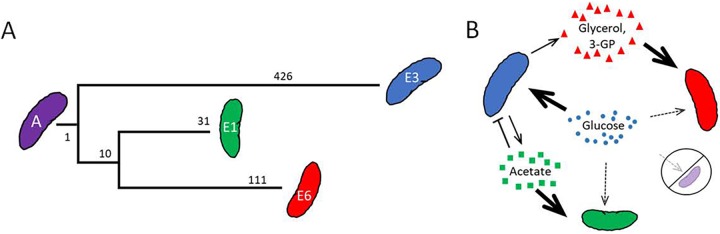
Evolution of a stable polymorphism. (A) Helling et al. (18) subjected the initially clonal population of E. coli strain JA122 (here, strain A) to hundreds of generations of continuous glucose limitation. At generation 773, a set of clones was isolated that were shown to be phenotypically distinct (Table 1) (18, 26, 27, 41). Strain A and its descendants could also be differentiated on the basis of hundreds of single-base-pair substitutions, including those in E3 that contribute to its glucose-scavenging, fermentative phenotype (*mglO*, *malK*, *hfq*, *ptsI*, and *lpd*), those in E1 that contribute to acetate scavenging (–93 *_p_acs*), and the retention of ancestral alleles in E6 (e.g., *ptsI*) that contribute to glycerol and glycerol 3-P assimilation (phylogeny redrawn from reference 8 [not to scale]). (B) Evolved clones coexist by cross-feeding, with the frequency of different clones dependent on the availability of the primary and secondary resources (26).

In the experiments described by Helling et al., stable polymorphism manifested as a set of four lineages distinguished by colony size and ampicillin resistance that stably coexisted over more than 800 generations of chemostat culture (18). At generation, 773 single colony isolates representative of these lineages were archived. Three of these isolates were later shown to form a consortium supported by cross-feeding between a primary resource specialist best able to scavenge limiting glucose and secondary resource specialists having the capacity to consume its overflow metabolites (Table 1 and Fig. 1) (8, 26, 27). The relationships between this primary resource specialist and the secondary specialists were complex, in one case apparently based on “resource-service” exchange (28, 29). Specifically, release of acetate by the glucose specialist provided carbon and energy for a secondary resource specialist that performed a “service” by scavenging this growth-inhibitory metabolite (8, 26, 27). The basis of this simple community appears to arise from a tradeoff between the capacity to acquire limiting substrate and the capacity to respire it completely, even in the presence of oxygen. The incidence of this kind of resource-service exchange in nature likely depends both on the particular mutations by which a primary resource specialist attains its status and on the nature of the primary resource (e.g., is it fermentable or nonfermentable carbon?).

**TABLE 1:** Strains used in this study and their relevant physiological properties

Strain	Strain characteristics	Mean uptake rate ± SEM		Mean growth on alternative carbon sources ± SEM^*c*^			
		Glucose^*a*^	Glycerol^*b*^	0.2% glucose		0.5% acetate	
				μ_max_	Yield	μ_max_	Yield
K-12 MG1655	F^–^ λ^–^ *ilvG rfb-50 rph-1*			0.31 ± 0.00	1 ± 0.01	0.00 ± 0.00	0.04 ± 0.00
JA122 (A)	F^–^ *thi-1 lacY1 tonA21 supE44 hss1 araD139*; lysogenic for λ, contains plasmid pBR322Δ5 (Amp^r^)	1.19 ± 0.09	99 ± 8	0.32 ± 0.01	1.15 ± 0.03	0.00 ± 0.00	0.02 ± 0.00
CV101 (E1)	Descendant of JA122; isolated after 773 generations of glucose-limited growth, Amp^r^, forms large colonies on TA.	1.66 ± 0.06	93 ± 7	0.38 ± 0.01	1.49 ± 0.02	0.02 ± 0.00	0.08 ± 0.00
CV103 (E3)	Descendant of JA122; isolated after 773 generations of glucose-limited growth, Amp^r^, forms small colonies on TA.	2.46 ± 0.16	104 ± 7	0.26 ± 0.00	1.08 ± 0.01	0.00 ± 0.00	0.00 ± 0.00
CV116 (E6)	Descendant of JA122; isolated after 773 generations of glucose-limited growth, forms small colonies on TA, lacks plasmid, Amp^s^	1.61 ± 0.11	146 ± 14	0.39 ± 0.00	1.27 ± 0.03	0.00 ± 0.00	0.01 ± 0.00

a The glucose uptake rate in batch culture is expressed as μmol of α-MG/min/g (dry weight) of biomass, as measured by Helling et al. (18).

b The glycerol uptake rate is expressed as pmol/min/10^8^ cells, as measured by Rosenzweig et al. (26).

c The maximum specific growth rate (μ^max^) is expressed as h^−1^. The cell yield was estimated as the optical density at λ = 550 nm and normalized to that of K-12 in Davis minimal medium containing 0.2% glucose. All values are means of three to four biological replicates.

Though microbes secrete myriad compounds, some as overflow metabolites (30–33), some as signaling molecules (34), and others as defensive or offensive weapons (35, 36), it is unclear how often these compounds become growth substrates for other microbes, be they members of the same taxon or different taxa. Moreover, even when they do, there may be evolutionary limits to the stability of cooperative interactions, owing to the risk of becoming dependent on a complementary ecotype and the possibility that cooperative metabolic exchanges reduce overall community productivity relative to a single generalist clone (37). To test for these possibilities and to deepen our understanding of conditions favoring evolution of a bacterial consortium from a single clone, we reexamined the cross-feeding population described by Helling et al. (18). We evaluated the competitive fitness of each consortium member, the fitness of pairs of strains, and the fitness of the consortium relative to fitness of their common ancestor, all under the original evolutionary conditions. We then related fitness estimates to estimates of resource consumption and productivity for each ecotype and for combinations thereof. We find that fitness and productivity increase in relation to functional complexity, measured as the number of coevolved ecotypes present in the system. Thus, just as syntrophic interactions among different taxa can increase the amount of energy extracted from complex environments in nature, cross-feeding interactions between adaptive lineages that arose from a common ancestor can increase the amount of energy extracted from a simple environment in the laboratory.

## RESULTS

### Coevolved consortia can be reconstructed in the laboratory.

A large, initially clonal microbial population often becomes polymorphic, with genetically distinct clades sometimes persisting over evolutionary time scales (18, 38–40). Early work by Helling et al. (18) showed that stable polymorphism can evolve in glucose-limited chemostats founded by a single E. coli clone. In one example, polymorphism took the form of evolved strains (CV101, CV103, and CV116) that differed in their colony size and antibiotic resistance, as well as in their capacity to scavenge limiting glucose (Table 1). Later studies revealed that the evolved clones had diverged both from their common ancestor (JA122 [here called strain A]) and from one another with respect to their proteomes (41), their transcriptomes (27), and their genome sequences (8, 42) (Fig. 1A). The persistence of lineages represented by these clones was shown to be driven by the evolution of a primary resource (glucose) specialist whose overflow metabolites (acetate, glycerol, and glycerol-3 phosphate) supported cross-feeding by secondary resource specialists (Fig. 1B) (26, 42). Whole-genome sequence data suggest that lineage-specific alleles confer the glucose, acetate and glycerol/glycerol 3-phosphate scavenging phenotypes (Fig. 1). A theoretical treatment of how primary and secondary resource specialists can evolve and coexist under continuous nutrient limitation has recently been developed by Gudelj et al. (43).

**FIG 2:**
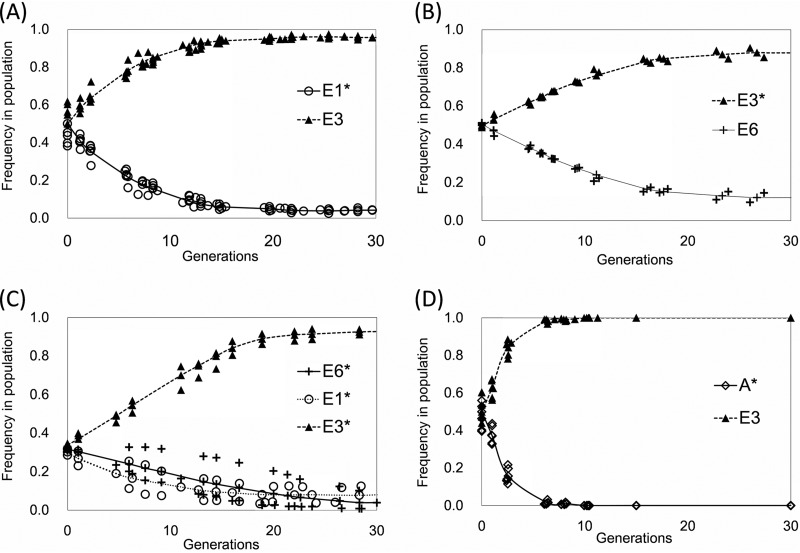
Reconstruction experiments. (A) E3+E1 consortium; (B) E3+E6 consortium; (C) E3+E1+E6 consortium. Three reciprocal experiments were performed so that in each experiment a single strain was GFP labeled and the frequency in the population was measured. (D) Displacement of the ancestor, A, by E3. Strain frequency in experimental populations was inferred by evaluating the frequency of FP-marked (*) strains by flow cytometry as described in Materials and Methods. Each data point represents a biological replicate.

To gain deeper insight into how and why cross-feeding consortia persist in chemostats we created green fluorescent protein (GFP)-tagged versions of the original Helling et al. E. coli strains (18). Flow cytometry data show that the consortium can be reestablished using GFP-labeled bacteria and that the relative frequency of consortium members agrees with prior studies where this parameter was estimated by scoring colony size and antibiotic resistance phenotypes (26). At steady state, the primary resource (glucose) specialist E3 is numerically predominant but stably coexists with either or both of two secondary resource specialists: E1, which has differential access to the overflow metabolite acetate, and E6, which has differential access to overflow metabolites glycerol and glycerol-3 phosphate (26; Julian Adams, unpublished data) (Fig. 2A to C). Secondary resource specialists E1 and E6 can also coexist, but not reproducibly, owing to the low frequency (<1%) of E1 cells in mixed E1:E6 populations at equilibrium (26). At steady state, the ratio of E3:E1 was approximately 9:1, whereas that of E3:E6 was approximately 5:1, which accords with previous estimates. In contrast, none of the evolved strains could stably coexist with their common ancestor. Each increased in relative abundance by 8 to 10% per generation, displacing the ancestor within 6 to 10 generations, as exemplified by E3 (Fig. 2D) and as summarized below.

### The fitness of evolved ecotypes is greater than that of their common ancestor, and the fitness of consortia is greater still.

**FIG 3:**
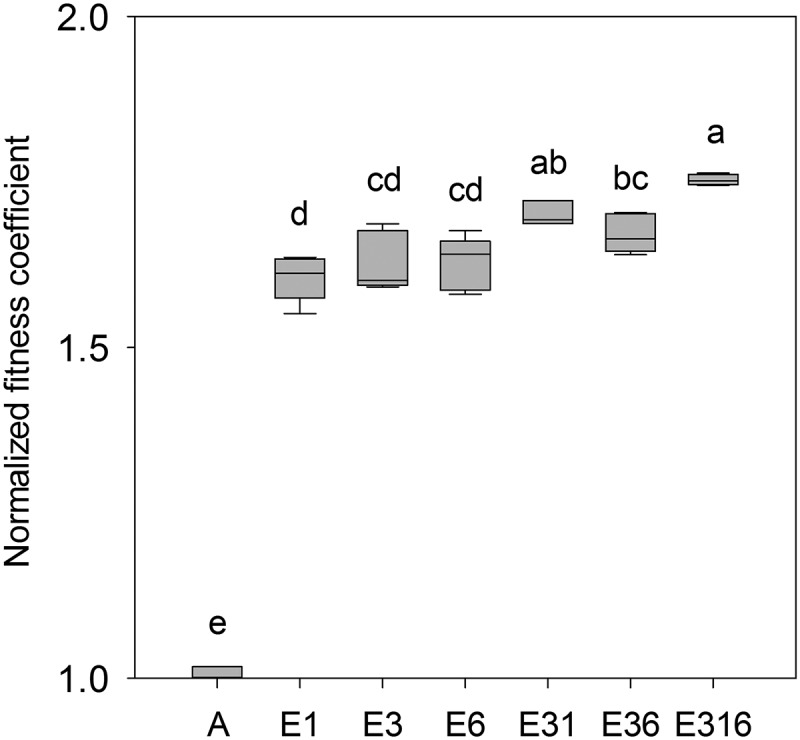
Normalized fitness coefficients of evolved strains relative to their common ancestor. Evolved strains E3, E1, and E6 or consortia (E3+E6, E3+E1, and E3+E6+E1) were competed with the GFP-labeled ancestor, A*. Significant differences were identified by one-way ANOVA, and the comparisons are indicated by lowercase letters. Nonidentical, lowercase letters indicate significance at *P* < 0.05 (Tukey’s HSD). The boundaries of the box and the whiskers correspond to the 25th and 75th percentiles and the 10th and 90th percentiles, respectively. Median lines are shown in boxes. Fitness estimates were generated from four to six independent biological replicates.

While the transcriptome and the whole-genome sequence of every strain in the evolved consortium have been described (8, 27), their fitness and productivity as individuals, and as collectives, have not. To estimate fitness, we first competed the GFP-labeled ancestral strain (A*) against the unlabeled ancestor (A) under evolutionary conditions (0.0125% glucose limitation). We found that the GFP-tagged strain had a slightly negative fitness coefficient (−0.076 ± 0.014). Thus, to compute relative fitness levels, we normalized competition coefficients to 1 for A versus A* to account for the fitness decrement arising from fluorescent protein (FP) expression, as we have done previously (44). We competed each evolved ecotype, as well as consortia composed of different ecotypes, against their common ancestor. Every evolved strain was significantly more fit than that ancestor (Fig. 3) (one-way analysis of variance [ANOVA] and Tukey’s honestly significant difference test [HSD], *P* < 0.01). Interestingly, evolved strains did not significantly differ from one another in fitness, relative to the common ancestor (one-way ANOVA and Tukey’s HSD, *P* > 0.9), even though they differed markedly from one another with respect to their glucose and glycerol uptake kinetics, as well as their growth rate and yield when batch cultured on different carbon sources (Table 1) (18, 26), their residual substrate concentrations (Table 2 and see Table S3 in the supplemental material) and in patterns of substrate utilization inferred from Biolog assays (see Fig. S1 in the supplemental material). Consortia consisting of two evolved ecotypes (E3+E1 and E3+E6) were more fit than their shared ancestor, and E3+E1 was more fit than E1, E3, or E6 monocultures (one-way ANOVA and Tukey’s HSD, *P* < 0.05) The three-membered consortium E3+E1+E6 was not only significantly more fit than the ancestor but also more fit than every evolved strain in monoculture, as well as the E3+E6 consortium (one-way ANOVA and Tukey’s HSD, *P* < 0.05). When directly compared by a paired *t* test, E3+E1+E6 was more fit than either E3+E1 or E3+E6 (*P* < 0.05).

**TABLE 2:** Residual steady-state metabolite concentrations

Strain	Mean metabolite concn (μM) ± SEM^*a*^			
	Glucose	Acetate	Formate	Ethanol
K-12 MG1655	2.00 ± 0.34^A^	77.12 ± 2.84^A^	0.00 ± 0.00^E^	0.00 ± 0.00^B^
A	1.33 ± 0.30^A^	54.43 ± 2.79^B^	18.91 ± 1.37^C,D^	0.06 ± 0.02^A^
E1	0.20 ± 0.03^B^	0.00 ± 0.00^D^	29.65 ± 0.50^A,B^	0.06 ± 0.03^A^
E3	0.09 ± 0.03^B^	36.85 ± 4.30^C^	32.89 ± 1.84^A^	0.11 ± 0.01^A^
E6	0.23 ± 0.06^B^	51.78 ± 2.70^B^	17.43 ± 1.06^D^	0.10 ± 0.03^A^
E31	0.10 ± 0.01^B^	0.00 ± 0.00^D^	21.92 ± 3.59^B^	0.08 ± 0.05^A^
E36	0.15 ± 0.02^B^	32.22 ± 2.06^C^	30.69 ± 1.77^A^	0.11 ± 0.02^A^
E316	0.18 ± 0.02^B^	0.00 ± 0.00^D^	27.26 ± 2.10^A,B,C^	0.00 ± 0.00^B^

a Values represent means of three to six biological replicates. Enzymatic assays were technically duplicated. Different superscript letters denote significant differences among strains and consortia at *P* < 0.05 by one-way ANOVA. Identical superscripts denote no significant difference.

### Relative fitness increases correlate with increases in productivity.

**FIG 4:**
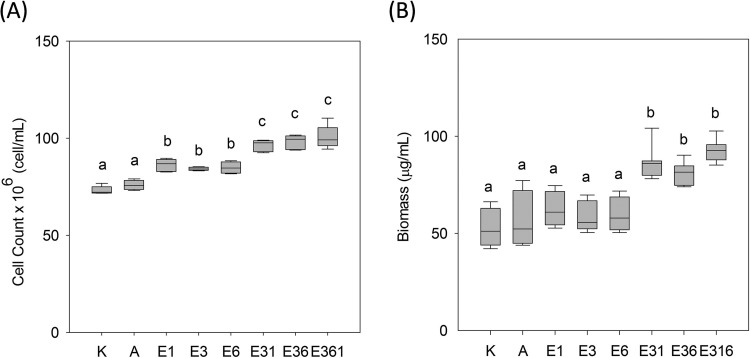
Productivity in steady-state chemostats of evolved strains and consortia relative to their common ancestor and to E. coli K-12 (K). (A) Yield expressed as cells ml^−1^; (B) yield expressed as dry-weight biomass in μg ml^−1^. Significant differences were identified using one-way ANOVA and are indicated by lowercase letters. Nonidentical lowercase letters indicate significance at *P* < 0.05 (Tukey’s HSD). The boundaries of the box and the whiskers correspond to the 25th and 75th percentiles and the 10th and 90th percentiles, respectively. Median lines were shown in boxes. All productivity estimates were generated from four to six independent biological replicates.

Next, we sought to determine whether the observed fitness differences among strains and consortia mapped onto productivity. The ancestor, the evolved ecotypes, and consortia of these ecotypes were each grown to steady state in replicate glucose-limited chemostats and then evaluated with respect to yield cell number and yield dry weight biomass per ml culture. So that our productivity estimates would be broadly comparable, we also cultured the canonical E. coli K-12 strain (MG1655) under the same conditions and included its yield values in our analyses. No significant differences in yield were detected between A and K-12 (Fig. 4), whereas each of the evolved strains (E1, E3, and E6) exhibited greater yield cell number than either A or K-12 (Fig. 4A) (*P* < 0.05, one-way ANOVA). When grown as monocultures, none of the evolved strains demonstrated a higher yield dry weight biomass than their ancestor (Fig. 4B), indicating that they had evolved smaller cell size, a frequent adaptation by bacteria to chronic nutrient limitation (45, 46). Cocultures of E3+E1 and E3+E6 produced higher yields than any monoculture (Fig. 4), with differences most pronounced when yield was estimated as dry weight biomass. The median value of biomass in chemostats containing three members, E3+E1+E6, was the highest of all tested and almost 2-fold greater than chemostats containing only A, the shared ancestor (Fig. 4B). In addition, we carried out Pearson correlation analysis of the mean values for fitness, biomass, and cell density for each of the evolved morphs and consortia. The Pearson correlation coefficients between fitness and biomass, between fitness and cell density, and between cell density and biomass are 0.94, 0.90, and 0.98, respectively. The analysis therefore indicates a positive correlation between the observed increases in fitness and the observed increases in productivity.

### Residual metabolite levels differ between monocultures and consortia.

When grown to steady state under continuous glucose limitation, the ancestor and the ecotypes that coevolved from that ancestor significantly differed with respect to concentrations of residual metabolites (Table 2 and Table S3). Differences in levels of residual glucose and acetate previously noted (26) among chemostat monocultures were largely confirmed here. Steady-state monocultures of K-12 or A had the highest levels of residual glucose, while those of evolved strain E3 had the lowest, <10% of the ancestral strain’s mean value, which is consistent with E3’s exceptional capacity to scavenge limiting glucose (Table 1). A one-way ANOVA uncovered no significant differences in residual glucose among monocultures of the evolved ecotypes or their consortia, though direct comparisons showed that residual glucose was lower in E3 than in either E1 or E6 (paired *t* test, *P* < 0.05). E1 left no residual acetate, confirming that it is an acetate scavenger (26, 43). Further, no residual acetate was detected in consortia containing the E1 ecotype (E3+E1 and E3+E1+E6), indicating that, when present, E1 completely consumes this secondary resource.

We also investigated whether other organic acids might be implicated in cross-feeding. Unlike E. coli K-12, formate overflow was observed in all single- and multistrain chemostat cultures (Table 2). Residual formate in E6 monocultures was comparable to that in A but was significantly less than in chemostat cultures of the other evolved ecotypes and the consortia (one-way ANOVA and Tukey’s HSD, *P* < 0.05). Residual ethanol was detected in all cultures but those of K-12 and the three-membered consortium, suggesting that, in the latter, one or more of its members either repressed ethanol-forming or activated ethanol-consuming pathways (Table 2). Residual 2-hydroxyglutarate (2-HG), the reduced product of tricarboxylic acid (TCA) intermediate 2-oxoglutarate, was manyfold higher in A monocultures than it was under any other culture condition. 2-HG was significantly lower in E3 than in any other monoculture, but it was indistinguishable from E3 in E3-containing consortia (Table S3). From this we conclude that E3 does not produce sufficient 2-HG for it to serve as a secondary resource and that the low levels of 2-HG observed in consortia likely arise from the fact that in these populations the frequency of E3 is ≥0.8 (Fig. 2). Why E3 has so strongly diverged from its ancestor in the excretion of 2-HG may be related to the fact that even under aerobic, glucose limitation the redox state of E3 is that of an anaerobic cell (43).

The TCA intermediate, *cis*-aconitate, was also significantly higher in chemostats populated by A than in those populated by K-12 or any of the other evolved ecotypes or their consortia. Five unknown metabolites were also identified by liquid chromatography-mass spectrometry (LC-MS) (Table S3). Most exhibited lower residual values in E3 monocultures and consortia than in monocultures of the ancestor, E1, E6, or K-12. As with 2-HG, these observations likely stem from the fact that at steady state the frequency of E3 in consortia is ≥0.8.

### Biolog assays reveals additional ways in which the experimentally coevolved strains are phenotypically differentiated.

To determine whether the evolved strains differed in other ways that create opportunities for metabolic interactions, we tested their substrate utilization patterns using the Biolog platform: a multiplexed phenotyping system carried out in 96-well format on batch cultured cells. Biolog phenotyping reinforces the view that the coevolved strains are ecotypes. Relative to the ancestor and to the other evolved strains, E1 grew best on acetate, pyruvate, malate, and fumarate (Fig. S4A), indicating a greater capacity for TCA cycle metabolism, as well as for aerobic and anaerobic respiration (Fig. S5). This observation is consistent with prior observations that ecotype E1 is especially well adapted to assimilating and respiring acetate as a secondary resource. Notable among those adaptations is constitutive overexpression of the low *K_m_* acetate-scavenging enzyme, acetyl coenzyme A (acetyl-CoA) synthetase due to an IS element insertion in the E1 *acs* promoter (8, 27). We also discovered multiple instances where evolved ecotypes differentially assimilated carbon, nitrogen, phosphorus, and sulfur growth substrates (Fig. S4A to D). For example, relative to E1 and E3, ecotype E6 grew best on diffusible intermediates such as 6-phosphogluconate, d-2-phosphoglyceric acid, β-glycerol phosphate, glycerol, and d,l-α-glycerol phosphate (Fig. S4C and D), indicating that ecotype E6 has greater assimilative capacity via the pentose phosphate and Entner-Doudoroff pathways (Fig. S5). These data bolster prior observations (8, 26, 43) that the secondary resources to which E6 has differential access include glycerol and glycerol-3 phosphate. While Biolog-based generalizations about organisms’ growth attributes are known to be problematic (see, for example, reference 47), and Biolog assays are performed on batch, not chemostat, cultures, the existence of so many complementary metabolic capacities, coupled with additional evidence that E6 is especially adept at using diffusible three-carbon intermediates, opens up possibilities for cross-feeding interactions not readily detected by assaying residual substrate levels in spent chemostat media.

## DISCUSSION

### Ecotypic differentiation and partnership in a simple constant environment.

When grown in isolation, the ancestral and coevolved strains described here differ with respect to their growth rate and yield in batch culture, their glucose uptake kinetics, their relative susceptibility to acetate inhibition, and their propensity to release or to take up a variety of overflow metabolites (18, 26, 43). Because these strain-specific physiological differences are associated with genetic differences across scores of loci (8), E1, E3, and E6 should be regarded as ecotypes that have undergone adaptive diversification (48). Continued genetic differentiation among these ecotypes has the potential to result in *bona fide* species, as envisioned by Fraser et al. (49) and others (17, 50).

All things being equal, when a group of organisms competes for a single limiting resource, the variant best able to scavenge that resource to the lowest concentration will prevail (51–53). Interestingly, the relative fitness of ecotype E3 was not greater than E1 or E6, even though it exhibited superior glucose uptake kinetics and consistently rose to highest frequency in consortia (26). E3 was originally isolated as a small colony variant and later shown to be a glucose specialist that opens up new niches by releasing overflow metabolites to which it has limited access (26). Ecotypic variants E1 and E6 exploit these, and the fitnesses of E3+E1, E3+E6, and E3+E1+E6 consortia all exceed fitness of the ancestor. Median fitness values for partnerships were greater than those of all monocultures, ancestral or evolved. That E3+E6 was not significantly more fit than either E3 or E6 in monoculture may be due to the fact that E6, unlike E1, does not consume an inhibitory overflow metabolite (acetate) but rather exhibits high affinity for noninhibitory metabolites such as glycerol-3 phosphate. Yield cell number among consortia exceeded that in evolved ecotypes, their common ancestor, and in E. coli K-12. This finding is consistent with theory indicating that there is a second way for an ecotype to prevail under simple nutrient-limiting conditions, namely, to become efficient at converting the limiting resource to offspring (54, 55). In this system, consortia have a double evolutionary advantage since the collective excels at both strategies: scavenging the limiting resource and converting that resource to offspring.

Ecotypes E3 and E1 exhibit a reciprocal interaction that takes the form of “resource-service exchange” (28, 29). E3 scavenges glucose to residual levels that are inaccessible to E1 but excretes a “resource,” overflow carbon in the form of acetate, to which E1 has preferential access. The E1 ecotype in turn provides a “service” to ecotype E3, by consuming this growth-inhibitory metabolite (56) to which E3 is especially sensitive (43). Under glucose limitation the fitness of E3+E1 consortia exceeds that of ecotypes E1 and E3, validating the conclusion that their coexistence is a form of mutualism that we have previously termed “clonal reinforcement” (8).

The role played by ecotype E6 is more subtle: E6 exhibits superior growth on three-carbon metabolites and amendment of glucose-limited E3+E6 consortia with glycerol or with glycerol-3 phosphate renders E6 numerically dominant over E3 (26; unpublished data). However, a resource-service interaction between E3 and E6 based on overflow glycerol or glycerol-3 phosphate may be difficult to achieve, since neither of these compounds is growth inhibitory. Since the relative fitness levels of E3 and E6 do not significantly differ, they may owe their coexistence to a special form of clonal interference, albeit one stabilized by cross-feeding of three-carbon exometabolites, a possibility we aim to test in future experiments. Whatever the mechanism, populations having the highest overall median fitness were those that were most complex, an observation consistent with a large body of literature showing positive correlations between community and ecosystem diversity, stability, and productivity (57–60).

### Ecological complexity in the microbial world stems from chemical interactions.

Microbes engage in every conceivable form of biotic interaction, ranging from intraspecific and interspecific competition (61, 62) to predation (63, 64), to a variety of cooperative interactions that encompass communal feeding (65, 66), to detoxification of growth-inhibitory compounds (67), and to cross-feeding (68, 69). Played out against a backdrop of spatiotemporal variation in abiotic factors such as pH, redox potential, pO_2_, inorganic ions, light, and temperature (70–77), biotic interactions give rise to the structure and dynamics of microbial communities in nature. Of the interactions that are cooperative, all are ultimately mediated by chemicals secreted into the extracellular environment, be they quorum-sensing molecules (78), degradative enzymes (79), polysaccharides (80), or overflow metabolites (81), including hydrogen (82).

Synthetic biology has proved to be a powerful tool for unraveling microbial interactions driven by secreted exometabolites (83, 84), leading for calls to standardize fabricated ecosystems in order to systematically dissect signaling and metabolic exchange in natural communities (85). For example, cross-feeding can be genetically engineered, leading to the discovery that in E. coli division of labor between one genotype that carries out glucose catabolism and others that consume overflow metabolites boosts productivity in batch, chemostat, and biofilm cultures (86). Similarly, the distribution of two anabolic pathways between an engineered yeast and an engineered E. coli mutualist enhanced natural product formation (87). Several groups have generated complementary amino acid auxotrophs and locked them into obligate mutualisms (88–91). These systems have been used to evaluate the fitness costs and stability of this type of microbial interaction and to discover the type of genetic changes that ensue once it is established (92, 93).

Notwithstanding insights offered via synthetic biology, few studies have reported on how cross-feeding spontaneously emerges in laboratory evolution experiments, especially those carried out under constant resource-limiting conditions. Models based on such studies open up the possibility for identifying genetic and ecological conditions in nature that favor cross-feeding (43, 94), a phenomenon that occurs in both clinical (95, 96) and industrial settings (97–99), with important consequences for therapy and bioproduction. Spontaneous evolution of cross-feeding within a species may even be a prelude to syntrophy, which by convention involves multiple species and which is most often seen in environments where electron acceptors are scarce and complex electron donors are fermented by one species to educts like H_2_, formate, or acetate and then oxidized by partner species (28, 100–104).

Theory suggests syntrophy may be rare, owing to inherent risks in obligate metabolic codependency, as well as to diminished efficiency of substrate use by a collection of strains relative to one that is autonomous (37, 105). Still, genetic diversity in several experimental systems has been attributed to coevolution of lineages pursuing complementary metabolic strategies (18, 26, 38, 39, 106). Thus, metabolic interactions in the form of cross-feeding, whether within or between species, likely play a key role in determining microbial community structure in nature (107).

### Could cross-feeding within a population of facultative anaerobes lead to syntrophy?

Syntrophy is regarded as an ecological strategy by which bacterial and/or archaeal partners couple their metabolism in a manner that increases the amount of energy extracted from scarce resources under anoxic conditions (28, 101, 103). By carrying out reaction sequences in different cellular compartments and then coupling them, a syntrophic consortium can make a thermodynamically unfavorable reaction favorable, as in the well-known example of ethanol fermentation coupled to methanogenesis (108). Here, experimental evolution of E. coli, a facultative anaerobe that can mineralize glucose (18), results in genetically differentiated subpopulations that compartmentalize this process. The cross-feeding interactions that sustain this consortium are based on one clone behaving like an anaerobe, even in the presence of oxygen.

Why be avid but wasteful when resources are limited, and why does this collective outcompete an autonomous generalist? Multiple hypotheses have been advanced to explain why bacteria and other cells achieve their maximum growth rate by switching from respiration to fermentation (109). One idea is that the complete oxidation of glucose via the TCA cycle results in overproduction of reduced cofactors, especially NADPH, and that the switch enables cells to adjust redox status (110, 111). Other hypotheses include economic arguments based on the cost of synthesizing TCA enzymes (112), electron transport proteins (113), and/or the possibility that (inner) membranes may become space limited for the integration of electron transport proteins (114). In any case, fermentation allows for faster ATP production per unit membrane area (109).

An adaptive phenotype shared by all consortium members is upregulation of outer and inner membrane proteins involved in glucose scavenging, notably LamB and the MglBAC complex (27). Transcripts for these membrane proteins are most dramatically overexpressed in E3 (27), which may place a premium on membrane “real estate” that could otherwise be dedicated to electron transport proteins. E3 harbors unique *de novo* mutations in lipoamide dehydrogenase (*lpd*) that reinforce its fermentative strategy (8). In fact, under aerobic conditions E3’s redox state is like that of an anaerobic cell (43), a state wherein enzyme-flux cost minimization models predict that yield-inefficient pathways like acetogenic fermentation provide a growth advantage (55). However, the glucose-avid E3 ecotype comes at a cost: it excretes overflow metabolites that it cannot respire, creating secondary resources for ecotypes that can (Fig. 1). In this example, coordinated metabolism via cross-feeding helps the consortium as a whole to maximize ATP production and yield, while simultaneously minimizing enzyme and intermediate concentrations (112, 115).

How pervasive is intercellular metabolic compartmentalization when resources are limiting? Evolution experiments are needed that compare the frequency with which cross-feeding arises under both anaerobic and aerobic conditions and where cells are limited on either fermentable or nonfermentable carbon sources. Equally informative will be experiments designed to test the effects of historical contingency. Specifically, are E3-specific mutations, such as missense mutations in multifunctional Lpd (lipoamide dehydrogenase) or E1-specific mutations in acetate scavenging enzyme Acs (acetyl-CoA synthetase) (8), required for cross-feeding to evolve in E. coli under continuous glucose limitation?

### Cross-feeding interactions are likely ubiquitous.

Syntrophic cross-feeding interactions exist not only between anaerobes but also between anaerobes and aerobes (116), plants, and microbes (117) and between animals and their gut microbiota (118, 119). Cross-feeding may even play a role in chronic microbial infection (120), since complementary auxotrophies have been observed during early phase Pseudomonas aeruginosa cystic fibrosis lung infections (121, 122). Whether polymorphic microbial populations in these “natural experiments” are collectively more fit and/or more productive than their ancestors, or one another in isolation, has yet to be determined. Cross-feeding is also a conspicuous feature of organisms that have differentiated multicellularity, with different nutritive functions segregated into different compartments. Lastly, cross-feeding also arises within genetically heterogeneous tumors (123–125), as well as between tumor cells and the surrounding normal tissue to which they are related (126, 127). Only now are researchers beginning to elucidate the genetics and demography of how these metabolic interactions arise and persist in cancer and in chronic infections, which may ultimately help to explain their resilience and their resistance to drug therapy (128). Lastly, the observation that genetically diverse microbial communities, whether engineered or arising spontaneously, exhibit higher fitness (90) and productivity (86) than those that are less so opens up the possibility of engineering novel communities that capitalize on cross-feeding to achieve complex and/or energetically difficult tasks in a stable, robust way (129–132).

## MATERIALS AND METHODS

### Strains, media, and culture conditions.

All E. coli strains (Table 1), including those fluorescent protein (FP)-tagged strains used in flow cytometry experiments, were archived as 20% glycerol stocks at −80°C. For brevity, ancestral strain JA122 is here designated “A,” and its evolved descendants CV101, CV103, and CV116 are designated E1, E3, and E6, respectively. Davis minimal medium [DMM; 7 g liter^−1^ K_2_HPO_4_, 3 g liter^−1^ KH_2_PO_4_, 1 g liter^−1^ (NH_4_)_2_SO_4_, 0.1 g liter^−1^, MgSO_4_⋅7H_2_O, 0.001 g liter^−1^ thiamine-hydrochloride] was used for all chemostat cultures with 0.025% glucose added for batch cultures and 0.0125% glucose added for chemostats (133). The maximum specific growth rates (μ_max_) presented in Table 1 were assayed at 30°C using a Synergy HTX multimode microplate reader (BioTek, Winooski, VT) using 48-well plates, each of which contained 0.5 ml of DMM amended with 0.025% glucose (wt/vol). μ_max_ was calculated by following the change in absorbance at λ = 550 nm over time and calculated as previously described (18).

Chemostat inocula were prepared from single colonies on tryptone agar (TA; 10 g liter^−1^ tryptone, 5 g liter^−1^ NaCl, 14 g liter^−1^ agar, with or without ampicillin) that were grown overnight at 30°C in liquid batch culture. Continuous culture was carried out using the Infors Sixfors bioreactor system (CH-4103; Infors AG, Bottmingen, Switzerland). Chemostats were aerated with sterile ambient air and maintained at 30°C at a dilution rate, D, of ≈0.2 h^−1^ for ∼70 h (∼15 culture generations). Cultures were sampled at prescribed intervals and assayed with respect to cell density by measuring absorbance at 550 nm and by counting the CFU following serial dilution and plating on TA. Colonies were further scored with respect to colony size (large or small) and antibiotic sensitivity (resistant or sensitive) as described previously (18, 134).

### Fluorescent protein labeling of *E. coli* strains.

To estimate the relative frequency of different E. coli strains as they were competed in chemostats, we monitored the change in frequency of GFP-tagged strains over successive generations. An expression cassette containing GFP under the constitutive PA1 promoter was integrated into the genomes of the ancestral and evolved strains using a modified Tn*7* delivery system (135). Details of pGRG36-Kan-GFP plasmid construction are described in the supplemental material and the primers used are listed in Table S2. Transposition of the GFP cassette to the attachment site (*attTn7*) at the 3′ end of *glmS* was confirmed by sequencing and epifluorescence microscopy. Our novel plasmid pGRG36-Kan-GFP, which enables rapid GFP tagging of either the E. coli, *Salmonella*, or *Shigella* chromosomes, is available from Addgene (catalog no. 79088; Addgene, Cambridge, MA). The same protocol was used to tag E. coli with other fluorescent proteins, specifically mCherry, cyan FP (CFP), and yellow FP (YFP). However, in follow-up experiments where a labeled ancestor was competed against an unlabeled ancestor, GFP was the only fluorescent marker that exhibited marginal effects on host strain fitness (coefficient = −0.076 ± 0.014). Strains bearing one of these other FPs had fitness coefficients much greater than −0.100. Thus, the data reported in Fig. 2 and 3 were generated using GFP-marked strains.

### Reconstruction experiments.

Evolved strains and their ancestor can be distinguished on the basis of colony size on TA and ampicillin resistance (Table 1). Earlier work using these phenotypes had shown that the evolved consortium could be reconstructed as various combinations of the original strains cryopreserved as −80°C glycerol stocks (26). Three groups of reconstruction experiments were carried out under previously described conditions (26), except that in each chemostat vessel one of the strains was GFP-labeled to enable its frequency to be monitored over time by flow cytometry. In pairwise reconstructions, strains were reciprocally marked, and the incidence of the GFP-labeled strain was monitored for at least 30 generations. Three-strain reconstructions were carried out as a set of independent experiments in which one GFP-labeled strain at a time was monitored, again for at least 30 generations. To confirm the presence of unmarked partners, colony size and ampicillin resistance were also routinely scored.

### Fitness estimates.

Relative fitness of each member of the evolved community, as well as various combinations of those members, was estimated under evolutionary conditions by competing them against the GFP-tagged version of their common ancestor, JA122 (denoted as A*). For each experiment, single colonies of A* and the strain(s) to be analyzed were suspended in 3 ml of DMM (0.025% glucose) and cultured overnight at 30°C with shaking at 150 rpm. Overnight cultures were inoculated into individual Sixfors bioreactors containing 300 ml of DMM (0.0125% glucose). Reactors were initially run as monocultures, operated at 30°C in batch mode for 8 to 10 h until the cell population attained an absorbance at 550 nm (*A*_550_) of 0.1, whereupon they were shifted to continuous mode at dilution rate D ≈ 0.2 h^−1^ and grown to steady state, undergoing approximately three volume changes.

To initiate competition between two strains, the labeled ancestor, A*, and one of its descendants (E1, E3, or E6), equivalent cell numbers from each steady-state monoculture were mixed in a 300-ml working volume bioreactor to produce a starting *A*_550_ of ∼0.1, the typical density achieved by strains at steady state under the limiting glucose concentrations used by Helling et al. (18). Once combined, which established time zero (*T*_0_), the reactor was placed under continuous glucose limitation and run at D ≈ 0.2 h^−1^ for 15 to 20 generations. To initiate competitions between the labeled ancestor, A*, and one of several possible multistrain consortia, monocultures of each evolved strain were first grown as described above and then mixed to reconstitute the consortium at previously described steady-state ratios (18, 26): E3+E1 = 5:1, E3+E6 = 4:1, and E3+E6+E1 = 5:1:1. Each consortium was cultured under continuous glucose limitation for 12 h (∼2 volume changes) before adding an equal number of the GFP-labeled ancestor, A*, to produce a *T*_0_ starting density of *A*_550_ ≈ 0.1. All competition experiments were performed under conditions identical to those under which the consortium had evolved. In both sets of experiments, the strain frequency dynamics were monitored by flow cytometry of chemostat samples that had been taken every 4 to 5 h, mixed with 0.5 ml of DMM containing 50% glycerol (vol/vol), and stored at −20°C until analysis.

### Flow cytometry.

To estimate relative abundance of GFP-labeled and unlabeled cells, chemostat samples were diluted 50-fold in 1% (wt/vol) NaCl and analyzed on an Attune NxT acoustic focusing flow cytometer (Thermo Fisher, Waltham, MA), with a minimum of 10,000 cell events per collected sample. A 50-mW, 488-nm laser was used to reveal scatter and fluorescence signals. The forward scatter (FSC) signal was detected with a photodiode detector, while the side scatter (SSC) and fluorescence signals were detected using photo multiplier tube detectors. GFP was detected on a 530/30 bandpass filter refined with a 503-nm long-pass dichroic filter. During instrument setup, the threshold was set in SSC, which is preferred for bacterial detection, rather than the typical FSC. Bacteria were first gated in the FSC/SSC dot plot to include the bulk of the cells, while excluding debris and large clumps. Single cells were gated and viewed in either a histogram or dot plot of fluorescence versus SSC. A gate was placed around the positive signal, and the resulting percentage was recorded. For controls, pure cultures of GFP-labeled and unlabeled E. coli JA122 were run to view the background fluorescence. The per-generation fitness coefficient was calculated as the slope of the linear regression ln(experimental/reference) as a function of elapsed generations; cell generations elapsed equals (time × dilution rate)/ln_2_ (44). Values were calculated as normalized means ± the standard deviations from at least four independent experiments. To verify the presence of each experimental strain and to check for contamination during the competition, culture samples were also plated on TA (with or without ampicillin) and scored for Amp^r^/Amp^s^ and large/small colony size as previously described (18).

### Cell count and biomass dry weight.

Yield parameters were estimated on individual strains and consortia grown to steady state (three-to-five volume changes) in 0.0125% (wt/vol) glucose-limited chemostats. The cell density per ml was estimated by diluting chemostat cultures 50-fold with 1% (wt/vol) NaCl and then subjecting the resulting suspension to flow cytometry, as described above. The per-ml cell number was estimated by the events per μl recorded in the gated region where debris and large clumps were excluded. The dry-weight biomass was estimated by filtering a 200-ml aliquot of cells onto preweighed 47-mm-diameter HNWP (hydrophilic nylon white plain) membranes (0.2-μm pore size; Millipore, Burlington, MA), drying the filters at 65°C for 24 h, and then reweighing them using a semi-micro-analytical balance (XPE26C, 1-μg readability; Mettler Toledo, Columbus, OH).

### Residual metabolites.

For each strain and consortium, 50 ml of medium was sampled at steady state from three to four independent chemostat runs. Each sample was filter sterilized first through a 0.2-μm HNWP membrane (Millipore) filter and then through a 0.22-μm cellulose nitrate syringe filter. Filtrates were stored in sterile 50-ml Falcon tubes at −80°C prior to analysis. Samples for LC-MS analysis were refiltered using a 0.2-μm PES sterile syringe filter and stored at −20°C.

### (i) Residual glucose.

Before assaying glucose, which is at a low concentration in steady-state chemostats, 10-ml portions of sterile filtrate were concentrated 20-fold by lyophilization and then resuspended in 0.5 ml of sterile Millipore water. Extracellular glucose was assayed enzymatically using a d-glucose kit (catalog no. 10716251035; Roche, Basel, Switzerland), based on the production of NADPH, which was assayed spectrophotometrically at 340 nm.

### (ii) Residual ethanol, acetic acid, and formic acid.

Concentrations of acetic acid and formic acid in culture supernatants were analyzed using an Agilent (Santa Clara, CA) 1100 HPLC system equipped with an Agilent Hi-Plex H column (7.7 by 300 mm) with 8-μm packing equipped with a guard cartridge (5 mm by 3 mm). A 20-μl volume of sample was injected, using 10 mM sulfuric acid as running buffer. The column compartment was held at 65°C, and the flow rate was 0.6 ml/min. Analytes were detected using a diode array detector set to 210 nm. Concentrations were determined by comparison to spiked-in standards. The residual ethanol concentration was determined enzymatically, as previously described (136).

### (iii) Other organic acids.

Cell supernatants were analyzed using acidic reversed-phase LC-MS on an Agilent Technologies 6540 Q-TOF, as previously described (137).

### Biolog phenotyping.

Utilization of 190 carbon sources, 95 nitrogen sources, 59 phosphorus sources, 35 sulfur sources, and 95 nutritional supplements was conducted by Biolog, Inc., phenotype microarray services (Hayward, CA) using the OmniLog system v1.5 (138). Eight 96-well plates, each containing 95 substrates and one negative well (PM1-8), were inoculated with either the A, E1, E3, or E6 strain and assayed in duplicate for 48 h at 30°C. Substrates for which the absolute value of the background-corrected average well height for test (E1, E3, or E6) minus the average well height for reference (A) strains for both replicates passed a static threshold determined by the company were considered differentially utilized. Positive differences were scored as gained phenotypes, and negative differences were considered phenotypic losses. Clustering of average well height values was done using the MeV TM4 software suite (available at https://sourceforge.net/projects/mev-tm4/).

### Data availability.

Plasmid pGRG36-Kan-GFP, which enables rapid GFP-tagging of the *E. coli*, *Salmonella*, or *Shigella* chromosomes, is available via Addgene (no. 79088). The ancestral *E. coli* strain JA122 and its evolved descendants CV101, CV103, and CV116 are available upon request from Frank Rosenzweig.

## Supplementary Material

Supplemental file 1
