# Supplementary material for: Fitness and Productivity Increase with Ecotypic Diversity among Escherichia coli Strains That Coevolved in a Simple, Constant Environment
Source: Appl Environ Microbiol. 2020 Apr 1;86(8):e00051-20. doi: 10.1128/AEM.00051-20 (PMC7117940; doi:10.1128/AEM.00051-20)

SUPPLEMENTARY INFORMATION

SUPPLEMENTARY MATERIALS S1: **Construction of GFP labeled *Escherichia coli* strains.** To develop a view of sub-population dynamics in the consortia and to estimate fitness of single strains and consortia, relative to their common ancestor, we monitored the change in frequency of Green Fluorescent Protein (GFP)-tagged *E. coli* by flow cytometry. The GFP expression cassette was integrated into the genome of the ancestor JA122 (A), as well as into evolved strains CV103 (E3), CV101 (E1), and CV116 (E6) using the Tn7 transposition delivery system described by McKenzie *et al* (1). Vector pGRG36 was obtained from Addgene and linearized with *Fsp*I (CR0135S, New England BioLabs Inc.), which disrupts the β-lactamase (bla)+ coding sequence. A kan^R cassette was then amplified from the pKIKOarsBKn vector (2) using primers P_FspI_Km_R and P_FspI_Km_R that generate 30-bp sequences homologous to the 5' and 3' ends of the kan^R cassette (see below **Table S1**), assembled with *Fsp*I linearized pGRG36 using the Gibson Assembly kit (E5510S, New England Biolabs Inc.), and transformed into *E. coli* strain DH5α for plasmid amplification and sequencing verification. The resulting plasmid was named pGRG36-Kn. Plasmid propagation followed protocols described by (1). The pSC101 origin, low-copy, and temperature-sensitive pGRG36 plasmid derivatives were propagated within the DH5α host at 30°C, then purified using the Zyppy™ Plasmid Midiprep Kit. If not otherwise stated, standard molecular biological procedures were performed.

Construction of the PA1 promoter for the GFP plasmid was accomplished via five rounds of PCR. PCR round #1 used primer P_gs_PA1_F (PAGE purified) and primer P_PA1_R to produce a sequence containing PA1 promoter and 30-bp assembly sequence. PCR #2 used primer P_pa1_GFP_F and primer P_ GFP_R to amplify the GFP coding sequence from vector

pJMBGFP. PCR #3 used primer P_ter_F and primer P_ter_R to amplify the terminator sequence from the pET-mCherry-LIC vector (Addgene, plasmid #29769). PCR #4 used primer P_gs_PA1_F and primer P_GFP_R to fuse the overlapping promoter sequence with the GFP CDS. PCR round #5 used primer P_gs_PA1_F and P_ter_R to fuse the full-length GFP expression cassette. The resulting PA1-GFP cassette was assembled with *Not*I linearized pGRG36-Kn using Gibson Assembly, transformed into *E. coli* DH5α, followed by Zyppy™ Midiprep, sequencing verification and named as pGRG36-Kn-PA1-GFP. Plasmid pGRG36-Kn-PA1-GFP was transformed into ancestral JA122 and descendent strains (CV101, CV103, and CV116). Transformants were plated on LB agar (containing 34 mg/L Kanamycin) and incubated for two days at 30°C to select for Tn7 transposon-mediated chromosome integration. Single colonies were then streaked on Luria agar and cultured at 42°C to cure the plasmid as per McKenzie et al. (1). GFP cassette transposition to the attachment site (*attTn7*) at the 3' end of *glmS* was verified by sequencing PCR products amplified from gDNA using primers P_*att*Tn7_F and P_*att*Tn7_R), and by checking fluorescence using epifluorescence microscopy.

References:

1. McKenzie GJ, Craig NL. Fast, easy and efficient: site-specific insertion of transgenes into Enterobacterial chromosomes using Tn7 without need for selection of the insertion event. *BMC Microbiology*. 2006;6.
2. Sabri S, Steen JA, Bongers M, Nielsen LK, Vickers CE. Knock-in/Knock-out (KIKO) vectors for rapid integration of large DNA sequences, including whole metabolic pathways, onto the Escherichia coli chromosome at well-characterised loci. Microb Cell Fact. 2013;12:60.

SUPPLEMENTARY MATERIALS S2:

Primers used in construction of GFP strains

Name	Sequence
P_FspI_Km_R	cgatgcctgcagcaatggcaacaacgttgcgccgcaagcactcagggcgc
P_FspI_Km_R	gagtaagtagttcgccagttaatagtttgcttgaagctggggtgggcgaa
P_gs_PA1_F	ttaattaatcagatcccgggtcaatagcggttatcaaaaagagtattgacttaaagt ctaacctataggatacttacagccatcgagagattaaagaggagaaaggcgaa
P_PA1_R	ttcgcctttctcctctttaatctctc
P_pa1_GFP_F	gagagattaaagaggagaaaggcgaa**ATG**AGTAAAGGAGAAGAACTTT
P _GFP_R	gcggatccttatggagttggtta**CTA**TTTGTATAGTTCATCCATG
P_ter_F	taaccaactccataaggatccgc
P_ter_R	cgtggcgcgcctcctaggtgctcgagtggcgtggatgtccggatatagtt
p_gs_Ter	cgtggcgcgcctcctaggtgctcgagtggcgtggatgtccggatatagtt
P_attTn7_F	gatgctggtggcgaagctgt
P_attTn7_R	gatgacggtttgtcacatgga

Underlined sequences correspond to the pGRG36 homologous sequences. Capitalized sequence is the 22 base pair overlapping sequence for fusion PCR, bold text indicates start and end of amplified GFP coding sequence.

SUPPLEMENTARY TABLE S3.

Relative abundance of secondary metabolites in filtrate of steady state chemostats.

**Strain**	**2-hydroxyglutarate**	**Aconitate**	**U210**	**U235**	**U242**	**U486**	**U575**
K	100.0±5.2^{c}	100.0±3.8^{bc}	100.0±6.8^{a}	100.0±7.2^{a}	100.0±6.7^{c}	100.0±31.6^{a}	100.0±3.6^{a}
A	572.1±65.7^{a}	196.7±13.3^{a}	111.4±6.7^{a}	75.1±5.4^{b}	462.8±32.8^{b}	1.5±0.9^{b}	76.7±6.7^{b}
E1	219.2±17.6^{b}	61.8±2.4^{bcd}	108.2±2.4^{a}	70.1±4.5^{b}	7530.6±890.0^{a}	1.1±0.3^{b}	98.9±4.9^{a}
E3	56.3±5.0^{c}	83.5±5.6^{c}	52.0±1.4^{b}	34.2±1.7^{c}	376.4±30.3^{b}	0.5±0.2^{d}	45.3±2.9^{c}
E6	119.4±13.4^{b}	48.3±3.2^{cd}	124.8±5.8^{a}	62.1±5.1^{b}	374.8±20.5^{b}	0.8±0.2^{c}	72.8±2.0^{b}
E31	77.4±7.6^{c}	56.0±11.9^{bd}	46.3±8.0^{b}	29.1±4.9^{c}	1392.0±398.1^{b}	0.4±0.2^{de}	40.8±6.5^{c}
E36	64.8±2.8^{c}	87.6±4.3^{bc}	61.4±2.6^{b}	37.2±1.6^{c}	321.1±11.8^{b}	0.5±0.1^{d}	51.9±1.7^{c}
E316	62.7±3.2^{c}	74.1±1.1^{bcd}	63.6±2.1^{b}	40.1±0.7^{c}	553.4±57.7^{b}	0.4±0.0^{ed}	56.4±3.9^{c}

Values represent the means ± SEM of at least 6 replicates. Different superscripts denote significant differences among strains and consortia at $P<0.05$ by one-way ANOVA. Identical superscripts denote no significant difference. Unknown metabolites are designated with U, followed by the m/z of the metabolite (M-H is the primary ionization mode under these conditions).

SUPPLEMENTARY FIGURES S4 A-D

BIOLOG assay of growth on different substrates. Heat maps depict growth for differentially utilized substrates as the average BIOLOG well height difference between evolved isolates and their common ancestor. Map scales were set to the minimum range necessary to include all values while maximizing visualization of strain differences. Negative values (blue) indicate lower relative respiration of a substrate while positive values (yellow) indicate enhanced relative respiration.

S4A. Carbon sources

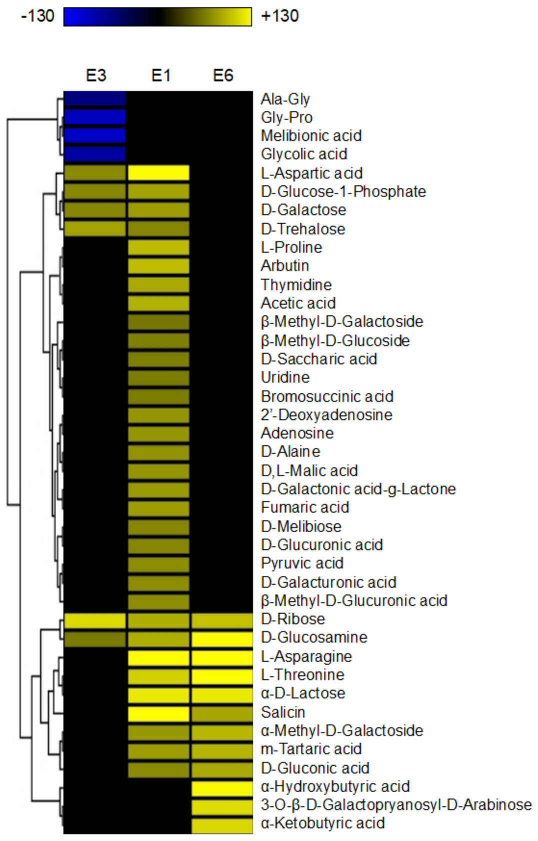

4B. Nitrogen sources

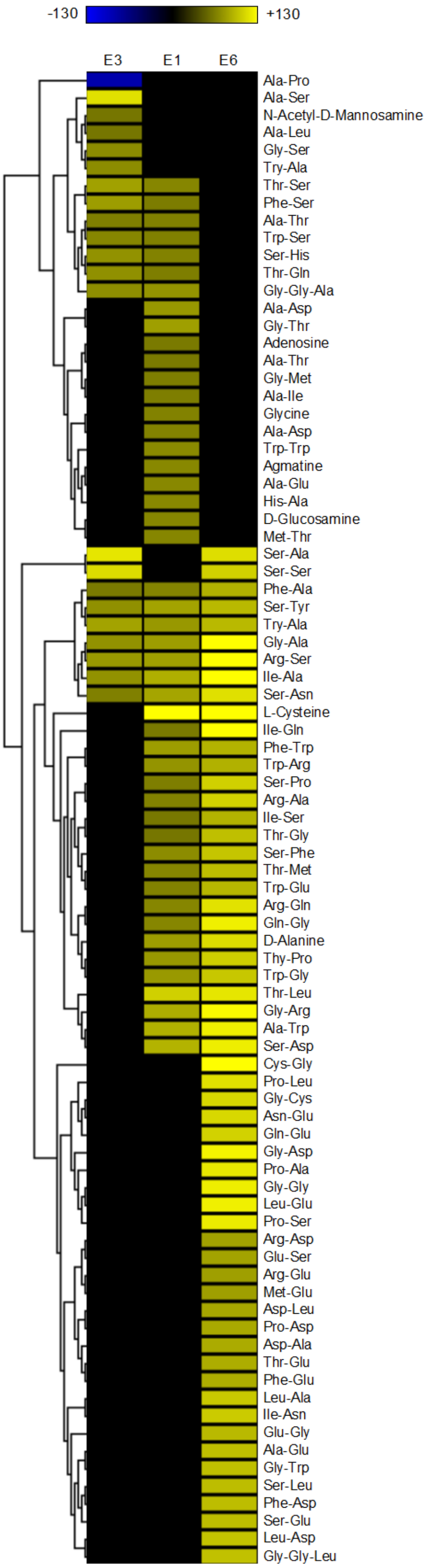

S4C. Nutritional supplements

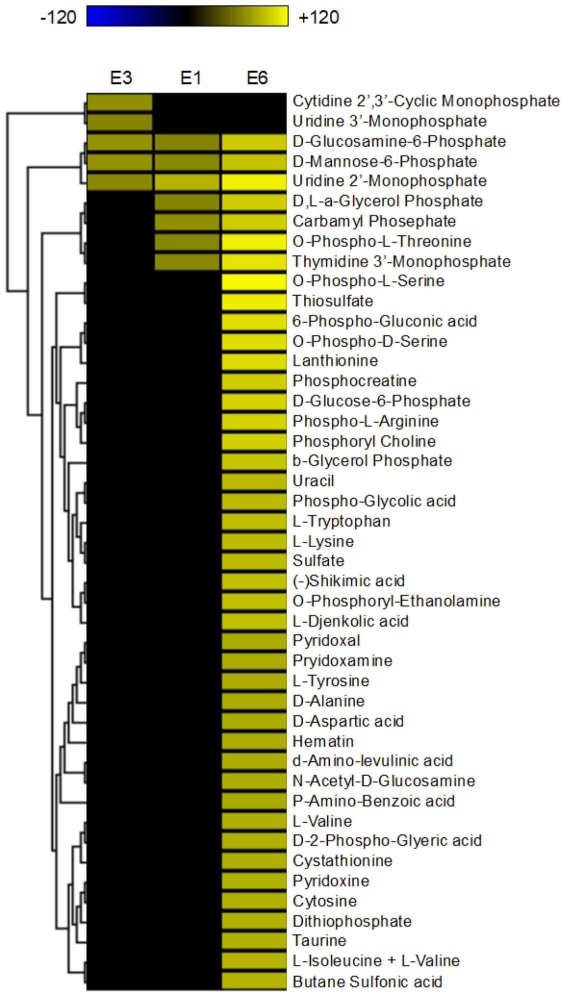

S4D. Phosphorus and Sulfur sources

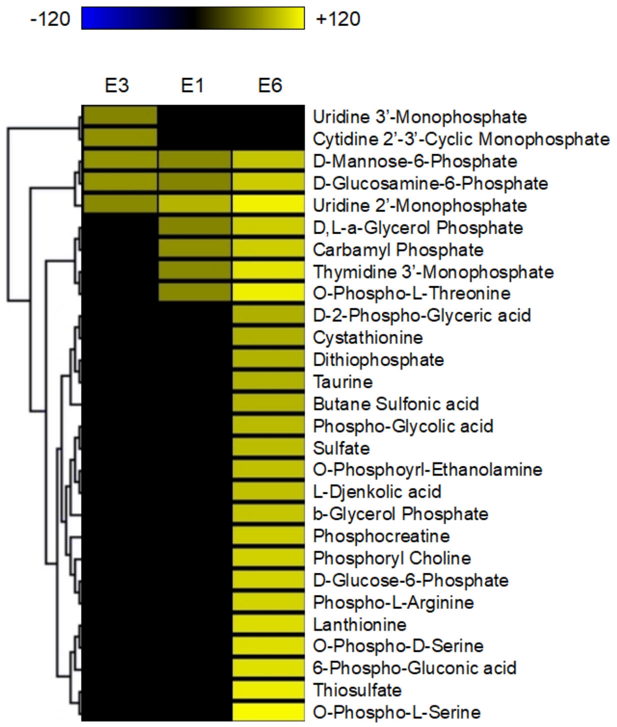

SUPPLEMENTARY FIGURE S5.

BIOLOG assay of growth on different substrates points to different strain-specific capacities to draw on multiple pathways. Small circles denote average well height difference for individual substrates and large circles represent the average difference across all identified substrates in a pathway.

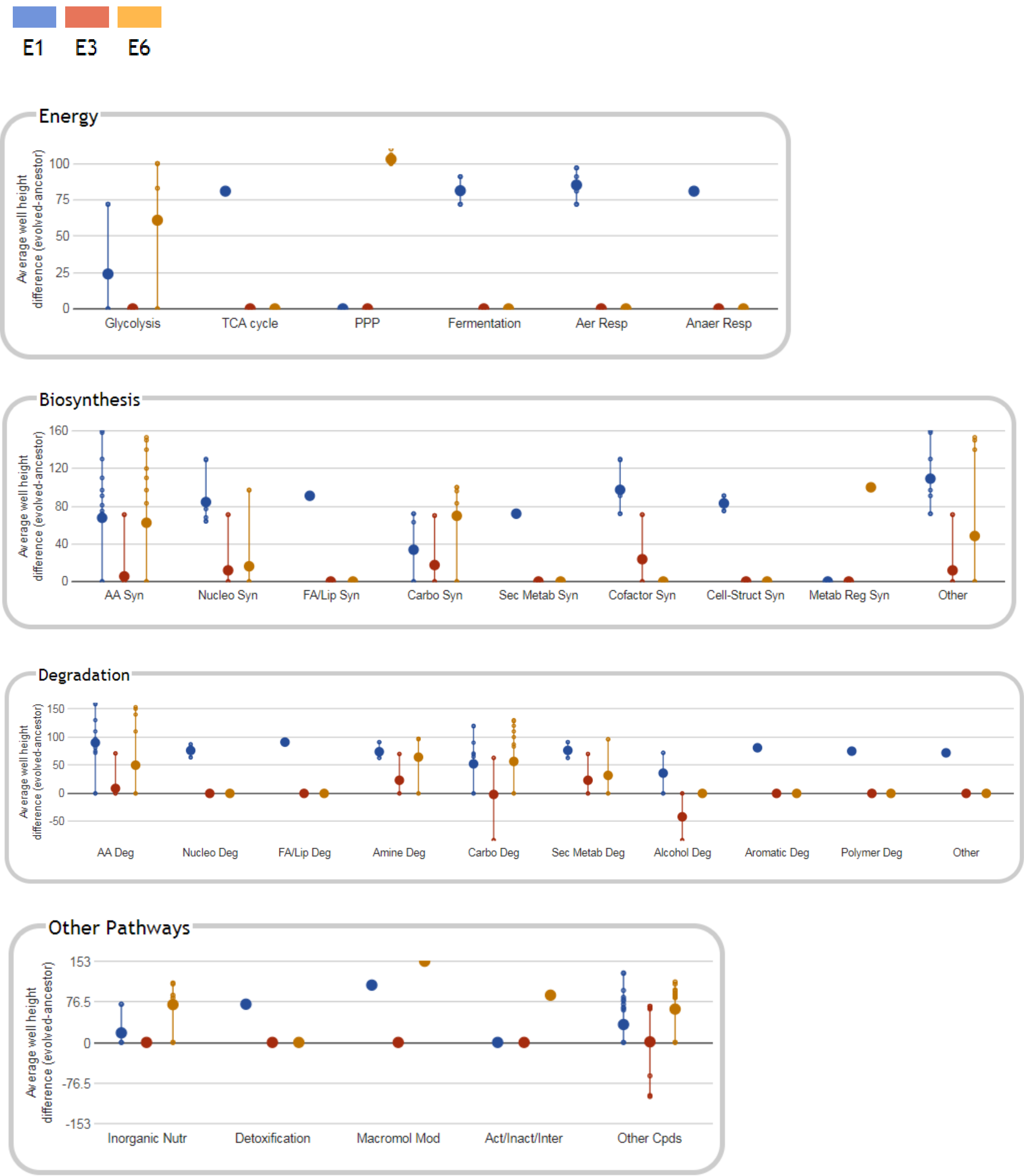

Supplement: Supplemental file 1 [file AEM.00051-20-s0001.pdf]
